# Perceptions and attitudes of dental students and dentists in South Korea toward artificial intelligence: a subgroup analysis based on professional seniority

**DOI:** 10.1186/s12909-024-05441-y

**Published:** 2024-04-22

**Authors:** Hui Jeong, Sang-Sun Han, Hoi-In Jung, Wan Lee, Kug Jin Jeon

**Affiliations:** 1 Department of Oral and Maxillofacial Radiology, Yonsei University College of Dentistry, Seoul, South Korea; 2 Department of Preventive Dentistry & Public Oral Health, Yonsei University College of Dentistry, Seoul, South Korea; 3 Department of Oral and Maxillofacial Radiology, Wonkwang University College of Dentistry, Iksan, South Korea

**Keywords:** Dentists, Dental students, Artificial intelligence, Perception, Attitude

## Abstract

**Background:**

This study explored dental students’ and dentists’ perceptions and attitudes toward artificial intelligence (AI) and analyzed differences according to professional seniority.

**Methods:**

In September to November 2022, online surveys using Google Forms were conducted at 2 dental colleges and on 2 dental websites. The questionnaire consisted of general information (8 or 10 items) and participants’ perceptions, confidence, predictions, and perceived future prospects regarding AI (17 items). A multivariate logistic regression analysis was performed on 4 questions representing perceptions and attitudes toward AI to identify highly influential factors according to position, age, sex, residence, and self-reported knowledge level about AI of respondents. Participants were reclassified into 2 subgroups based on students’ years in school and 4 subgroups based on dentists’ years of experience. The chi-square test or Fisher’s exact test was used to determine differences between dental students and dentists and between subgroups for all 17 questions.

**Results:**

The study included 120 dental students and 96 dentists. Participants with high level of AI knowledge were more likely to be interested in AI compared to those with moderate or low level (adjusted OR 24.345, *p* < 0.001). Most dental students (60.8%) and dentists (67.7%) predicted that dental AI would complement human limitations. Dental students responded that they would actively use AI in almost all cases (40.8%), while dentists responded that they would use AI only when necessary (44.8%). Dentists with 11–20 years of experience were the most likely to disagree that AI could outperform skilled dentists (50.0%), and respondents with longer careers had higher response rates regarding the need for AI education in schools.

**Conclusions:**

Knowledge level about AI emerged as the factor influencing perceptions and attitudes toward AI, with both dental students and dentists showing similar views on recognizing the potential of AI as an auxiliary tool. However, students’ and dentists’ willingness to use AI differed. Although dentists differed in their confidence in the abilities of AI, all dentists recognized the need for education on AI. AI adoption is becoming a reality in dentistry, which requires proper awareness, proper use, and comprehensive AI education.

## Background

Artificial intelligence (AI) enables machines to perform tasks with a level of intelligence comparable to that of humans. It has been approximately 10 years since AI passed through 2 periods of stagnation, often referred to as “winters,” and it has now entered its third heyday. AI is currently advancing faster than ever before, and commercialization has begun to occur in various fields. Translation, voice recognition, and navigation services have already been commercialized and are extensively used. Autonomous driving vehicles can be seen on the roads, and ChatGPT, a large language model, has recently come into the spotlight and is gaining worldwide popularity.

In dentistry, AI is applied to various image data, such as panoramic, periapical, bitewing, cephalometric, cone-beam computed tomography, and magnetic resonance images. AI has been studied extensively and can now be used across the entire process of dental care, from patient diagnosis to treatment plan establishment and prognosis evaluation. Dental AI is capable of diagnosing dental caries [1] and periodontal disease [2], identifying orthodontic landmarks [3, 4], predicting the difficulty of extracting wisdom teeth [5], and even accurately assessing patients’ facial asymmetry before and after orthognathic surgery [6]. As a report published in 2020 suggests [7], dental AI enables quick completion of tasks, accurate diagnosis through rational decision-making, and standardization of procedures. It is expected to become more customized to meet user needs and provide convenience.

School programs have begun to implement education on AI, and it is likely that dental students and seasoned dentists will become users of AI in the near future. The key will be individual choices about appropriate AI utilization and the technology’s reliability. To ensure the correct development and practical use of dental AI, a survey to understand the perceptions and attitudes of all generations of dentists must occur first. This approach makes it possible to identify whether students, with only theoretical knowledge, and dentists, with practical experience, align or differ in their opinions on dental AI based on their respective backgrounds. Some studies have been conducted in Brazil [8], Saudi Arabia [9], Turkey [10], and multinational institutions [11]. However, there has been no such attempt in South Korea; although some previous studies have analyzed the responses of dental students by their year of study [10], no studies have accounted for dentists’ length of professional experience.

Therefore, the aim of this study was to investigate and compare the current perceptions, confidence, predictions, and perceived future prospects of AI among dental students and dentists in South Korea. In addition, we categorized participants into subgroups to investigate whether their perceptions differed by their school year or length of professional experience.

## Methods

### Participant recruitment

This study was approved by the Institutional Review Board of the College of Dentistry, Yonsei University (No. 2–2022-0035). The sample size was calculated using G*Power software (ver. 3.1.9.7; Universität Kiel, Germany) with an effect size of 0.2, significance level of 0.05, and power of 0.85. An online survey using Google Forms was distributed from September to November 2022. Dental students were recruited from two randomly selected colleges (one in a metropolitan city and another in a rural area) out of a total of 11 dental colleges in South Korea. Dentists were recruited from the two most actively used dental social websites in South Korea: Dentphoto (http://www.dentphoto.com) and moreDEN (http://www.moreden.co.kr). Anyone enrolled in a Korean dental college or had a Korean dentist’s license could participate in the study. However, given that the major dental courses and clinical training begin in the third year in the 6-year Korean dental curriculum, recruitment was limited to students who were in their third year or higher. All participants confirmed their understanding and agreement to participate in the study by reading a research statement that was prepared in advance and completing the questionnaire.

### Questionnaire

The study used a comprehensive questionnaire developed by Jeong et al. [12] to survey all dental providers’ (dentists, dental hygienists, dental technicians, and students majoring in these fields, etc.) perceptions and attitudes toward AI. The reliability of the questionnaire was shown by a Cronbach’s alpha coefficient of 0.695 in the previous study, and minor details were modified to target dentists and dental students. The survey was distributed in their native language, Korean. It contained a basic section to investigate participants’ general information (8 questions for students, 10 for dentists), followed by a main section with 4 subsections to evaluate participants’ perceptions (5 questions), confidence (3 questions), predictions (4 questions), and perceived future prospects (5 questions) regarding AI. In the main section, participants could choose only one answer for each question, except in 3 multiple-response questions (items 9, 10, and 12). For the multiple-response questions, the number of possible responses was limited to determine priorities, with 2 for question 9 (“Which role will AI play in dental healthcare?”), 2 for question 10 (“Which field of dentistry will benefit most from AI?”), and 3 for question 12 (“Which branch of dentistry will be the first to commercialize AI?”). Participants were allowed to provide personalized responses if their opinion differed from the options presented, excluding questions on the ordinal scale.

### Data analysis

All responses were thoroughly anonymized before analysis. Frequency analysis was performed for all questions, and the questionnaire’s 5-point Likert scale was simplified to a 3-point scale to assess participants’ responses as a positive or negative tendency. The results of the frequency analysis are presented as percentages relative to the total number of participants (not the total number of responses). A logistic regression analysis was conducted to investigate the impact of multivariable factors (position, age, sex, residence, and self-reported knowledge level about AI of respondents) on perceptions and attitudes towards AI. One representative question was selected for each subsection of the main section, and all variables were classified into two categories for analysis. The two-tailed chi-square test was performed to analyze the statistical significance between dental students and dentists, and within the respective subgroups of dental students and dentists for the 17 main questions. Dental students were divided into lower years (third and fourth) and upper years (fifth and sixth), and dentists were divided into 4 groups according to how long ago they had received their dentist license: 5 years or less, 6–10 years, 11–20 years, and 21 years or more. The Fisher’s exact test was applied for questions where the chi-square test was not possible due to an expected frequency of fewer than 5 (exceeding 20% of the total). The statistical analysis was performed using SPSS version 26 (IBM Corp., Armonk, NY, USA), with a statistical significance threshold of *p <* 0.05.

## Results

### Participant recruitment

A total of 264 responses were collected, including 120 dental students (55.6%) and 96 dentists (44.4%). Data from 48 participants were excluded because they disagreed to participate in the survey or selected more responses than allowed (for instance, choosing 3 or more options when only 2 were allowed). General information about the participants is shown in Table 1. Among dental students, fourth-year students accounted for the most responses (35.0%). Dentists with more than 21 years of experience accounted for 31.3, and 81.3% of dentists worked in dental clinics. A total of 60.8% of dental students and 43.8% of dentists accessed the latest dental news through their schools or academic conferences. Approximately one-quarter of respondents (24.2% of dental students and 25.0% of dentists) stated that they well known or very well known about AI.

**Table 1:** Participants’ general information

Question	Dental students	Dentists
	N(%)	
**1. Which of the following applies to you?**		
Dental student	120(55.6)	–
Dentist	–	96(44.4)
**2. How old are you?**		
**≤** 30 years old	111(92.5)	12(12.5)
31–40 years old	9(7.5)	30(31.3)
41–50 years old	0	33(34.4)
51–60 years old	0	16(16.6)
**≥** 61 years old	0	5(5.2)
**3. What is your sex?**		
Male	77(64.2)	67(69.8)
Female	43(35.8)	29(30.2)
**4. Which of the following is your main residence?**		
Seoul	48(40.0)	53(55.2)
Capital area	13(10.8)	25(26.0)
Regional metropolitan city	22(18.4)	5(5.2)
Local neighborhood	33(27.5)	9(9.4)
Town or township	4(3.3)	4(4.2)
**5. Where do you usually find the latest dental news?**		
Internet	41(34.2)	36(37.5)
Newspapers	0	5(5.2)
Acquaintances	0	4(4.2)
Books and papers	6(5.0)	8(8.3)
Regional metropolitan cities	0	0
Schools or academic conferences	73(60.8)	42(43.8)
Other	0	1(1.0)
**6. How well do you know AI?**		
Very well / well	29(24.2)	24(25.0)
Average	47(39.2)	36(37.5)
Little / not at all	44(36.6)	36(37.5)
**7. Have you ever taken a part in AI development?**		
Yes, I have	7(5.8)	6(6.2)
No, I have not	113(94.2)	90(93.8)
**8. (Dental students only) Which year are you in?**		
3rd years	9(7.5)	–
4th years	42(35.0)	–
5th years	33(27.5)	–
6th years	36(30.0)	–
**9. (Dentists only) How long have you been licensed as a dentist?**		
**≤** 5 years	–	27(28.1)
6–10 years	–	15(15.6)
11–20 years	–	24(25.0)
**≥** 21 years	–	30(31.3)
**10. (Dentists only) Which is your dental specialty?**^**☨**^		
None (general dentist)	–	34(35.4)
Advanced general dentistry	–	37(38.5)
Basic dentistry	–	0
Conservative dentistry	–	0
Oral and maxillofacial radiology	–	3(3.1)
Oral and maxillofacial surgery	–	7(7.3)
Oral medicine	–	3(3.1)
Orthodontics	–	6(6.3)
Pediatric dentistry	–	3(3.1)
Periodontics	–	5(5.2)
Preventive dentistry	–	0
Prosthodontics	–	5(5.2)
*TOTAL*	–	*103(107.2)*
**11. (Dentists only) Which type of workplace do you work in?**		
Dental clinic	–	78(81.3)
(Dental) hospital	–	4(4.2)
(Dental) university hospital	–	11(11.4)
Enterprise	–	0
Public institution	–	3(3.1)
Educational institution	–	0
Other	–	0

*AI* artificial intelligence, *N* number of responses

^☨^Multiple-response question

### Questionnaire analysis

The results of the main section to evaluate participants’ perceptions, confidence, predictions, and perceived future prospects regarding AI are shown in Table 2.

**Table 2:** Items regarding AI, consisting of participants’ perceptions, confidence, predictions, and perceived future prospects

Question	Dental students	Dentists
	N(%)	
**Perceptions toward AI**		
**1. Are you interested in AI that is used in everyday life?**		
Strongly agree / agree	76(63.3)	56(58.3)
Neither agree nor disagree	32(26.7)	32(33.3)
Strongly disagree / disagree	12(10.0)	8(8.4)
**2. What is your main source to obtain AI information?**		
Internet	100(83.3)	74(77.1)
Newspapers	3(2.5)	5(5.2)
Acquaintances	4(3.4)	4(4.2)
Books or papers	1(0.8)	1(1.0)
Schools or academic conferences	10(8.3)	11(11.5)
Other	2(1.7)	1(1.0)
**3. What is the greatest advantage of AI?**^**b**^		
Fast and objective	22(18.3)	31(32.3)
Integration of extensive data	63(52.6)	26(27.1)
Reduction of misdiagnosis rates	22(18.3)	30(31.3)
No constraints of time and space	3(2.5)	0
No emotional exhaustion or physical limitations	10(8.3)	7(7.3)
Other	0	2(2.0)
**4. What is the greatest disadvantage of AI?**		
Difficult to use for controversial issues	19(15.8)	9(9.4)
Difficult to control unexpected situations	26(21.7)	27(28.1)
Lack of flexibility for individual applications	43(35.8)	29(30.2)
Limited consideration of the patients’ feelings	21(17.5)	23(24.0)
Developed by experts with little clinical experience	11(9.2)	5(5.2)
Other	0	3(3.1)
**5. Do you think that schools should provide education on AI?**		
Strongly agree / agree	51(42.5)	47(49.0)
Neither agree nor disagree	48(40.0)	39(40.6)
Strongly disagree / disagree	21(17.5)	10(10.4)
**Confidence in AI**		
**6. Do you think AI’s diagnostic ability could outperform skilled dentists?**		
Strongly agree / agree	30(25.0)	24(25.0)
Neither agree nor disagree	58(48.3)	42(43.8)
Strongly disagree / disagree	32(26.7)	30(31.2)
**7. Which would you trust more if yours and the AI’s judgment are different?**		
My judgment	59(49.2)	62(64.6)
AI’s judgment	11(9.2)	7(7.3)
Opinions of other dentists	41(34.2)	24(25.0)
Opinions of other AI programs	1(0.8)	1(1.0)
Leave it to the patient’s choice	8(6.6)	2(2.1)
**8. Who should be responsible when AI misdiagnoses?**		
Dentist	82(68.3)	73(76.0)
Dental hygienist	2(1.7)	0
Company that developed AI	24(20.0)	18(18.8)
Patient who followed AI’s opinion	11(9.2)	4(4.2)
Other	1(0.8)	1(1.0)
**Predictions about the application of AI**		
**9. Which role will AI play in dental healthcare?**^**☨**^		
Not helpful for dental healthcare	3(2.5)	0
A guide to solving rare problems	26(21.7)	15(15.6)
Providing clinical data for an evidence-based dental approach	49(40.8)	38(39.6)
Complementing the limits of human intelligence	73(60.8)	65(67.7)
Reference for each treatment	63(52.5)	30(31.3)
Complete replacement of dentist’s judgment	0	1(1.0)
Other	3(2.5)	2(2.0)
*TOTAL*	*217(180.8)*	*151(157.2)*
**10. Which field of dentistry will benefit most from AI?**^**☨**^		
Diagnosis	89(74.2)	82(85.4)
Treatment decision	42(35.0)	36(37.5)
Direct treatment including surgery	23(19.2)	11(11.5)
Dental care support for undeserved population	13(10.8)	9(9.4)
Research and development of drugs and dental materials	33(27.5)	20(20.8)
Development and improvement of social insurance system	7(5.8)	4(4.2)
Other	4(3.3)	0
*TOTAL*	*211(175.8)*	*162(168.8)*
**11. Which type of dental facility will be the first to commercialize AI?**		
Public primary care institution (e.g. public health center)	8(6.7)	9(9.4)
Primary care institution (e.g. private clinic)	14(11.6)	12(12.5)
Specialty clinic (e.g. orthodontics, esthetic prosthetics)	41(34.2)	30(31.3)
University hospital	57(47.5)	45(46.8)
Other	0	0
**12. Which branch of dentistry will be the first to commercialize AI?**^**☨**^		
Advanced general dentistry	8(6.7)	2(2.1)
Basic dentistry	34(28.3)	28(29.2)
Conservative dentistry	14(11.7)	12(12.5)
Oral and maxillofacial radiology	84(70.0)	68(70.8)
Oral and maxillofacial surgery	37(30.8)	20(20.8)
Oral medicine	25(20.8)	13(13.5)
Orthodontics	63(52.5)	47(49.0)
Pediatric dentistry	0	0
Periodontics	3(2.5)	2(2.1)
Preventive dentistry	9(7.5)	8(8.3)
Prosthodontics	24(20.0)	17(17.7)
Other	1(0.8)	1(1.0)
*TOTAL*	*302(251.6)*	*218(227.0)*
**Perceived future prospects for the application of dental AI**		
**13. Do you expect the application of dental AI to be useful?**		
Strongly agree / agree	90(75.0)	68(70.8)
Neither agree nor disagree	25(20.8)	23(24.0)
Strongly disagree / disagree	5(4.2)	5(5.2)
**14. When will dental AI be commercially available?**		
**≤** 3 years	6(5.0)	3(3.1)
4–7 years	35(29.2)	35(36.5)
8–11 years	50(41.6)	42(43.7)
12–15 years	17(14.2)	9(9.4)
**≥** 16 years	12(10.0)	7(7.3)
**15. How often would you use dental AI if it were applied?**^**a**^		
In all cases / in most cases	49(40.8)	35(36.4)
In about half of the cases	36(30.0)	18(18.8)
Only when absolutely necessary / seldom used	35(29.2)	43(44.8)
**16. Can dental AI replace your job in the future?**		
Strongly agree / agree	9(7.5)	6(6.2)
Neither agree nor disagree	34(28.3)	21(21.9)
Strongly disagree / disagree	77(64.2)	69(71.9)
**17. Will further improvements reduce misdiagnosis rates by dental AI?**^**b**^		
Strongly agree / agree	105(87.5)	77(80.2)
Neither agree nor disagree	10(8.3)	18(18.8)
Strongly disagree / disagree	5(4.2)	1(1.0)

*AI* artificial intelligence, *N* number of responses

^☨^Multiple-response question

^a ^Chi-square test; ^b^ Fisher’s exact test; *p* < 0.05

#### Perceptions toward AI

A total of 63.3% of dental students and 58.3% of dentists expressed an interest in AI. Only 8.3% of dental students and 11.5% of dentists responded that they had obtained information about AI from schools or academic conferences, and 42.5 and 49.0% of them, respectively, agreed that dental schools should provide educational programs on AI.

#### Confidence in AI

A total of 25.0% of both groups agreed or strongly agreed on the diagnostic superiority of AI over a skilled dentist. Only 9.2% of dental students and 7.3% of dentists answered that they would follow the AI’s judgment if it differed from their own. In cases when AI misdiagnosed a patient, 68.3% of dental students and 76.0% of dentists responded that the dentist should be responsible.

#### Predictions about the applications of AI

Both dental students (60.8%) and dentists (67.7%) selected “complementing the limits of human intelligence” as one of the top roles for AI in future dental healthcare, with some personalized responses such as “revenue analysis” or “patient attraction.” They predicted that dental AI would be useful in diagnosing diseases (74.2% of dental students and 85.4% of dentists). The majority of participants (70.0% of dental students and 70.8% of dentists) responded that oral and maxillofacial radiology would be the branch where AI would be commercialized first, followed by orthodontics (52.5% of dental students and 49.0% of dentists).

#### Perceived future prospects for the application of dental AI

A total of 75.0% of dental students and 70.8% of dentists answered positively to its potential utility. Many respondents predicted the commercialization of dental AI within 8 to 11 years (41.6% of dental students and 43.7% of dentists). Regarding the possibility that dental AI could replace their job in the future, 64.2% of dental students and 71.9% of dentists disagreed.

#### Multivariate factor analysis influencing perceptions and attitudes toward AI

The respondents who self-reported high level of knowledge about AI were more likely to show interest in AI than those with moderate and low level of knowledge (aOR = 24.345, *p* < 0.001) in multivariate factor analysis of perceptions and attitudes. There were no statistical significance depending on position, age, sex, and residence (Table 3).

**Table 3:** Analysis result of representative questions regarding AI-related perceptions, confidence, predictions, and perceived prospects according to the multivariable factors

		Perceptions			Confidence			Predictions			Perceived prospects		
		High interest in AI (Q1)			Performance superiority of AI (Q6)			AI commercialization at university hospital (Q11)			High usefulness of AI (Q13)		
		aOR	95% CI	*p*	aOR	95% CI	*p*	aOR	95% CI	*p*	aOR	95% CI	*p*
**Position**	Dental student	1.644	0.562–4.809	0.364	0.612	0.211–1.775	0.366	1.378	0.544–3.490	0.499	1.500	0.531–4.237	0.444
	Dentist	(ref.)											
**Age**	≤ 30	0.769	0.266–2.222	0.627	1.878	0.647–5.452	0.246	0.720	0.286–1.812	0.486	0.794	0.284–2.226	0.662
	> 30	(ref.)											
**Sex**	Male	1.605	0.856–3.012	0.140	1.785	0.869–3.669	0.115	0.703	0.391–1.264	0.239	1.037	0.539–1.994	0.914
	Female	(ref.)											
**Residence**	Seoul	0.883	0.473–1.647	0.696	0.851	0.448–1.619	0.624	1.397	0.804–2.427	0.235	1.033	0.555–1.922	0.918
	Other areas	(ref.)											
**Self-reported** **knowledge level about AI**	High	24.345	5.669–104.541	< 0.001^*^	0.973	0.467–2.027	0.941	1.163	0.610–2.217	0.647	1.340	0.632–2.841	0.445
	Not high	(ref.)											

*AI* artificial intelligence, *aOR* adjusted odds ratio, *CI* confidence interval, *p* p-value, *ref*. reference category

Logistic regression analysis; *p* < 0.05

### Difference analysis in perceptions and attitudes between dental students and dentists

Statistically significant differences were found between dental students and dentists for 3 questions in the questionnaire’s main section. In question 3, which asks about the greatest advantage of AI, dental students (52.6%) most often chose the integration of extensive data, while dentists (32.3%) most often responded that it is fast and objective. In question 15, dental students (40.8%) said they would use dental AI in all or most cases in the future, and dentists (44.8%) said they would use it only seldom or when necessary. Question 17 showed that a higher proportion of dental students (87.5%) than dentists (80.2%) anticipated that dental AI will develop and reduce misdiagnosis rates in the future (Table 2).

### Difference analysis within subgroups according to seniority of dental students and dentists

**Fig. 1:**
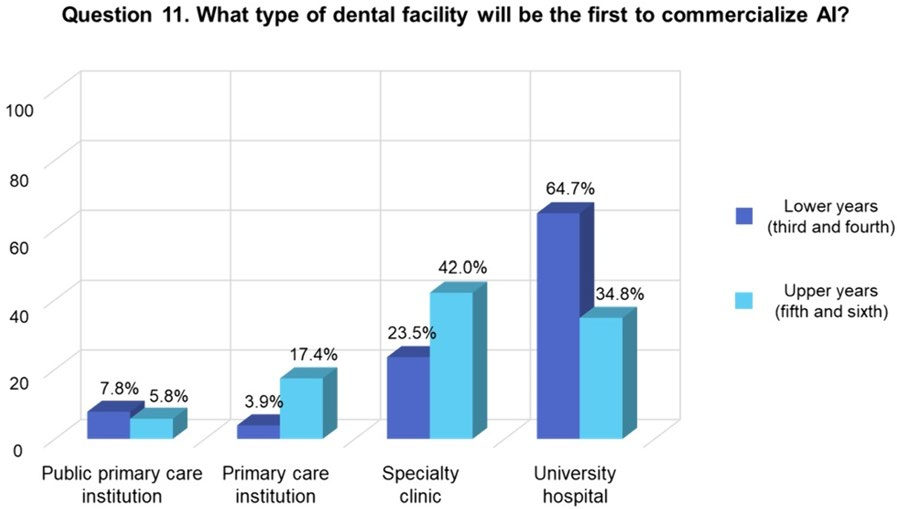
Responses of the student subgroups (lower and upper years) to Question 11. Fisher’s exact test showed a statistically significant difference in the responses of the 2 subgroups (*p* = 0.003). *AI* artificial intelligence

The subgroup analysis according to the seniority of dental students indicated that only the responses to question 11 exhibited a statistically significant difference (Fig. 1). Dental students in the lower years (third and fourth) believed (64.7%) that university hospitals would be the first to commercialize AI, while 42.0% of students in the upper years (fifth and sixth) thought that specialty clinics would be the first to do so.

**Fig. 2:**
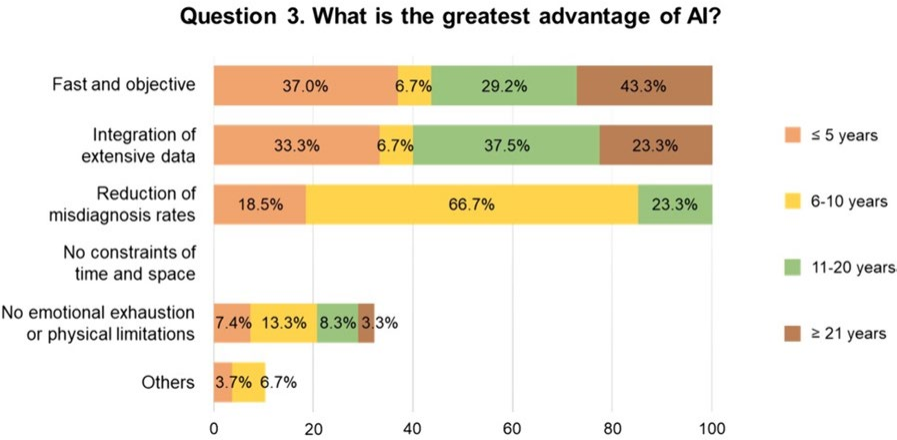
Responses to Question 3 according to dentists’ years of experience. Fisher’s exact test showed a statistically significant difference in the responses of the subgroups (*p* = 0.029). *AI* artificial intelligence

**Fig. 3:**
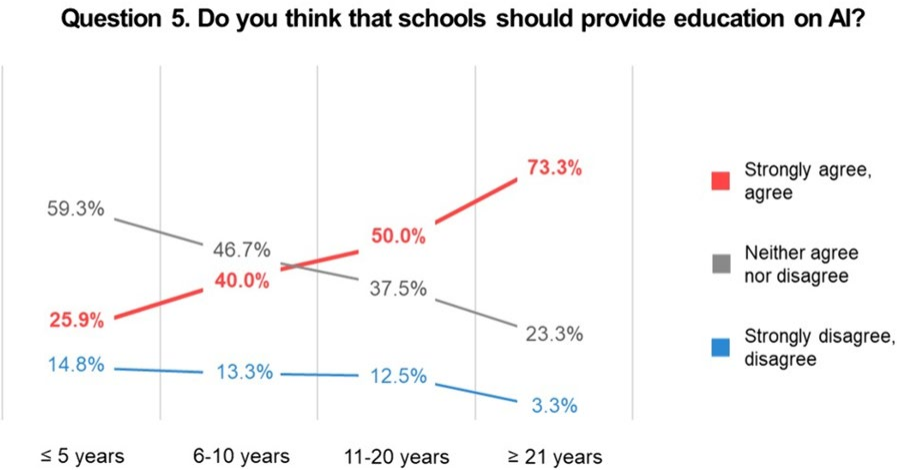
Responses to Question 5 according to dentists’ years of experience. Fisher’s exact test showed a statistically significant difference in the responses of the subgroups (*p* = 0.020). *AI* artificial intelligence

**Fig. 4:**
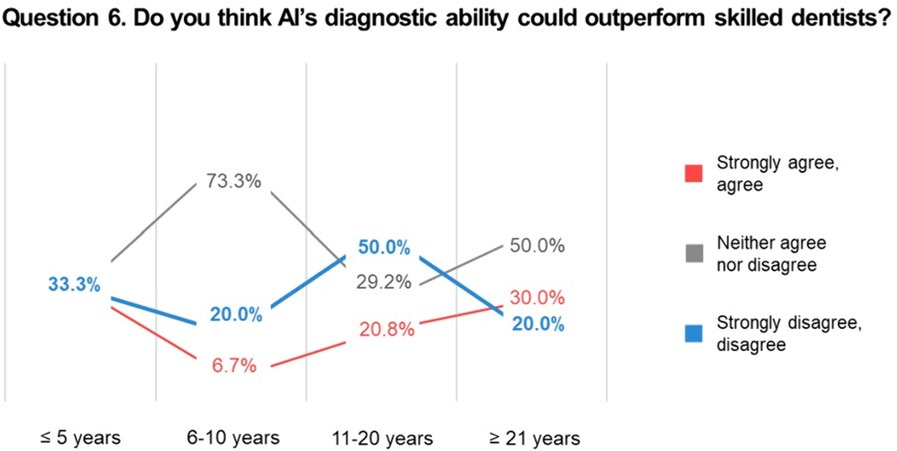
Responses to Question 6 according to dentists’ years of experience. The chi-square test showed a statistically significant difference in the responses of the subgroups (*p* = 0.043). *AI* artificial intelligence

In the subgroup analysis according to dentists’ seniority, 3 questions exhibited statistically significant differences. The most frequently mentioned strength of AI in question 3 was its fast and objective nature among the subgroup with 21 years or more of experience (43.3%), followed by the subgroup with less than 5 years of experience (37.0%). The subgroup with 6–10 years of experience chose “reduction of misdiagnosis rates” (66.7%), and the subgroup with 11–20 years chose “integration of extensive data” (37.5%). None of the dentists selected “no constraints of time and space” (Fig. 2). Question 5 indicated that the longer the dentists’ careers, the more they agreed that AI-related information should be provided in schools: 25.9% for 5 years or less, 40.0% for 6–10 years, 50.0% for 11–20 years, and 73.3% for 21 years or more (Fig. 3). In question 6, 50.0% of dentists with 11–20 years of experience disagreed that AI’s diagnostic ability could outperform that of a skilled dentist (Fig. 4).

## Discussion

The field of dentistry is undergoing significant changes through extensive research and development in dental AI, and its use is becoming a consideration across the dental profession, not only for a select few. This study aimed to investigate the perceptions and attitudes of dental students and dentists toward AI in South Korea. Some studies have been previously conducted, but this is the first attempt in South Korea. The novelty of this research lies in not only comparing the results between dental students and dentists but also analyzing differences based on their seniority. Furthermore, it is expected that the rapid development of AI will lead to changes in perception, so it would be interesting to compare the results of our study with the perceptions described in previous studies.

This study included 216 participants, consisting of 120 dental students and 96 dentists. The response rate for students was 20.7%, but an accurate calculation was not possible for dentists because the number of subscribers was not disclosed on the websites. Several strategies suggested by Phillips et al. [13] were implemented to collect as many responses as possible, but the possibility of non-response bias due to the small number of participants must be considered. The findings revealed no significant differences among dental students and dentists in most of their perceptions, confidence, predictions, and perceived future prospects of AI. Interestingly, despite the rapid advancement of AI technology, the overall trend observed in this study remains consistent with previous studies [8–11] conducted 2 to 3 years earlier.

In a previous survey of medical and dental students in 63 countries [11], only 15.3% of participants responded that they were interested in the use of AI in daily life, but our study observed a significant increase, with 63.3 and 58.3% of dental students and dentists, respectively. Nevertheless, it appeared that AI-related education is not yet sufficiently provided in schools. Only a small number of dental students and dentists answered that they obtained information on AI from schools or academic conferences (8.3 and 11.5%, respectively), and almost half of the participants (42.5 and 49%, respectively) responded that school programs should provide education on AI. Since several dental colleges have begun offering AI-related school programs, the proportion of participants who stated that schools should provide education on AI was somewhat lower than that in previous studies [8, 10, 11], and our results showed that related educational support is still lacking.

The findings in this study highlight the ethical challenges facing AI. Respondents said they would rely on the judgment of humans, such as themselves or other dentists, when their opinions and those of AI differed (83.4% of students and 89.6% of dentists). Although there was a prevalent belief that dentists should bear responsibility for AI misdiagnoses (68.3% of students and 76.0% of dentists), others pointed toward the companies that developed the algorithms (20.0% of students and 18.8% of dentists). AI algorithms have a blind spot, referred to as the “black box” problem, wherein they do not reveal the patterns they analyze and apply when learning from training data [14]. The opacity of AI decision-making processes and the potential inconsistency in judgments call into question the validity of AI, sparking debates on responsibility attribution [15]. Therefore, indiscriminate use of AI should be avoided in high-risk healthcare settings where accurate decisions and appropriate actions based on evidence are important.

Both students and dentists were aware of the role and limitations of AI. They expected AI to serve primarily as an adjuvant in diagnosing oral diseases (74.2 and 85.4%, respectively) and recognized that it could compensate for deficiencies in human intellectual limits (60.8 and 67.7%, respectively). More than 70% of participants identified oral and maxillofacial radiology as the field with the highest potential for dental AI to be commercialized first, followed by orthodontics. AI technologies, including machine learning and deep learning, are being utilized to accurately detect [16–20] or segment [21–23] oral lesions on dental radiographs. Several commercial software applications in orthodontics automatically recognize landmarks in this way.

Most students and dentists participating in our study had a positive outlook on the potential and utility of dental AI but did not believe it would replace their jobs (64.2 and 71.9%, respectively). Most agreed that AI cannot replace all jobs but expected that it will first replace simple jobs that do not require much skill. However, recent generative AI tools can create text, photos, and videos, and the idea that white-collar jobs based on professional knowledge will be replaced by AI is growing.

Respondents who considered themselves to have a high level of knowledge about AI were approximately 24 times more likely to be highly interested in everyday AI applications. There were differences in perceptions and attitudes toward AI depending on position, age, sex, and residence, but these were not statistically significant. It is thought that further research on this is needed in the future.

Contrasting opinions were observed between dental students and dentists on certain topics. First, dental students (52.6%) highly rated AI’s capability to integrate extensive datasets, but dentists (32.3%) prioritized AI’s fast and objective ability to identify abnormalities or diseases that are difficult to detect with the naked eye in real-world clinical settings. Second, students (40.8%) expressed an inclination to use dental AI actively, whereas dentists (44.8%) indicated a more selective approach to its usage. This discrepancy likely reflects differences between students who lack clinical experience and take theoretical classes and dentists who have substantial clinical experience; furthermore, most of the students were younger than 30 years old (92.5%), suggesting a greater ability to accept new concepts and the accompanying social changes.

When the results of the survey were compared between the upper- and lower-year subgroups of dental students, the differences in responses to all but 1 question were not statistically significant. Regarding the dental facilities where AI is anticipated to be commercialized first, lower-year students (64.7%) opted for university hospitals, while upper-year students (42.0%) chose specialty clinics such as orthodontics and aesthetic prosthetics. This difference may have been influenced by the students’ respective educational levels in dentistry.

The differences in perceptions and attitudes according to dentists’ seniority were more noteworthy. Dentists with more experience were more likely to agree that schools should provide AI-related education. This indicates that even older dentists unfamiliar with digital advancements now recognize the need for AI in the field. Now that the need for AI-related school programs is no longer a controversial issue, it is time to develop appropriate educational programs, including programs that can provide AI-related information to dentists in the field. Another difference was found in the responses to whether participants thought that AI’s diagnostic ability could outperform that of a skilled dentist. Dentists with less than 5 years of experience showed the same response rates (33.3% each) for agreement and disagreement, and 50.0% of dentists with 11–20 years of experience disagreed. Half of the dentists with more than 21 years of experience expressed neither agreement nor disagreement. Dentists with 11–20 years of experience are usually considered to be in their prime regarding their knowledge and diverse clinical experience, and they seemed to trust skilled dentists more than AI.

This study is significant in that it is the first study in South Korea to investigate overall perceptions and attitudes toward AI among dental students and dentists and the first in the world to analyze differences according to participants’ years of professional experience. The findings provide valuable insights into the challenges that AI researchers must address and directions for the application of AI to dentistry. Moreover, it may help with understanding the requirements of future and current dentists to use dental AI effectively in their practice. However, this study has some limitations. The sample size was small, and the data may have been biased since the participants were recruited from a limited number of dental colleges and social websites in South Korea. Additionally, this study did not include specific questions about dental AI, making it difficult to fully understand participants’ in-depth perceptions or attitudes. Lastly, the emergence of highly trained AI systems such as ChatGPT using large-scale data is significantly changing the paradigm and becoming increasingly integrated into healthcare, but this study was conducted prior to these developments. Given the rapid development of AI, there is a need for future surveys to track changes in perceptions over time and provide detailed analysis results.

## Conclusions

This survey showed that the greatest influential factor on perceptions and attitudes toward AI was the level of knowledge about AI, but the general view was found to be largely similar among dental students and dentists. Both dental students and dentists responded that they would follow their own judgment if it differed from that of an AI application and that dentists were responsible for AI-based diagnostic errors. This suggests that there is a growing tendency to perceive AI as an aid rather than to use it with blind faith. However, educational programs on AI are still lacking, and dentists with more experience showed the highest response rate regarding the need for AI-related education in schools. Students showed more active intention to use AI, and dentists expressed their intention to use it more selectively. These results showed that although some differences in perception still separate dental students and dentists by background and generation, the use of dental AI has become a reality for all dental students and dentists, and AI training for all generations is necessary.

## Data Availability

All data generated or analysed during this study are included in this published article.

## Abbreviations

AI: Artificial intelligence
ref.: Reference category
aOR: Adjusted odds ratio
CI: Confidence interval
*p*: *p*-value

## References

[1] Cantu AG, Gehrung S, Krois J, Chaurasia A, Rossi JG, Gaudin R (2020). Detecting caries lesions of different radiographic extension on bitewings using deep learning. J Dent..

[2] Danks RP, Bano S, Orishko A, Tan HJ, Moreno Sancho F, D’Aiuto F (2021). Automating periodontal bone loss measurement via dental landmark localisation. Int J Comput Assist Radiol Surg..

[3] Mahto RK, Kafle D, Giri A, Luintel S, Karki A (2022). Evaluation of fully automated cephalometric measurements obtained from web-based artificial intelligence driven platform. BMC Oral Health..

[4] Bulatova G, Kusnoto B, Grace V, Tsay TP, Avenetti DM, Sanchez FJC (2021). Assessment of automatic cephalometric landmark identification using artificial intelligence. Orthod Craniofac Res..

[5] Yoo J-H, Yeom H-G, Shin W, Yun JP, Lee JH, Jeong SH (2021). Deep learning based prediction of extraction difficulty for mandibular third molars. Sci Rep..

[6] Lin H-H, Chiang W-C, Yang C-T, Cheng C-T, Zhang T, Lo L-J (2021). On construction of transfer learning for facial symmetry assessment before and after orthognathic surgery. Comput Methods Prog Biomed..

[7] Tandon D, Rajawat J, Banerjee M (2020). Present and future of artificial intelligence in dentistry. J Oral Biol Craniofac Res..

[8] Pauwels R, Del Rey YC (2021). Attitude of Brazilian dentists and dental students regarding the future role of artificial intelligence in oral radiology: a multicenter survey. Dentomaxillofac Radiol..

[9] Abouzeid HL, Chaturvedi S, Abdelaziz KM, Alzahrani FA, AlQarni AAS, Alqahtani NM (2021). Role of robotics and artificial intelligence in Oral health and preventive dentistry-knowledge, perception and attitude of dentists. Oral Health Prev Dent..

[10] Yüzbaşıoğlu E (2021). Attitudes and perceptions of dental students towards artificial intelligence. J Dent Educ..

[11] Bisdas S, Topriceanu C-C, Zakrzewska Z, Irimia A-V, Shakallis L, Subhash J (2021). Artificial intelligence in medicine: a multinational multi-center survey on the medical and dental students' perception. Front Pub Health..

[12] Jeong H, Han SS, Kim KE, Park IS, Choi Y, Jeon KJ (2023). Korean dental hygiene students’ perceptions and attitudes toward artificial intelligence: an online survey. J Dent Educ..

[13] Phillips AW, Reddy S, Durning SJ (2016). Improving response rates and evaluating nonresponse bias in surveys: AMEE guide no. 102. Med Teach..

[14] Zednik C (2021). Solving the black box problem: a normative framework for explainable artificial intelligence. Philos Technol..

[15] Price WN (2018). Big data and black-box medical algorithms. Sci Transl Med..

[16] Ha E-G, Jeon KJ, Kim YH, Kim J-Y, Han S-S (2021). Automatic detection of mesiodens on panoramic radiographs using artificial intelligence. Sci Rep..

[17] Abu El-Ela WH, Farid MM, Abou E-FM. The impact of different dental restorations on detection of proximal caries by cone beam computed tomography. Clin Oral Investig. 2022;26:2413–20. 10.1007/s00784-021-04207-w 34601634

[18] Chen G, Huang L-G, Yeh P-C (2022). Detecting calcified pulp stones in patients with periodontal diseases using digital panoramic and periapical radiographies. J Dental Sci..

[19] Li S, Liu J, Zhou Z, Zhou Z, Wu X, Li Y (2022). Artificial intelligence for caries and periapical periodontitis detection. J Dent..

[20] Setzer FC, Shi KJ, Zhang Z, Yan H, Yoon H, Mupparapu M (2020). Artificial intelligence for the computer-aided detection of periapical lesions in cone-beam computed tomographic images. J Endod..

[21] Choi H, Jeon KJ, Kim YH, Ha E-G, Lee C, Han S-S (2022). Deep learning-based fully automatic segmentation of the maxillary sinus on cone-beam computed tomographic images. Sci Rep..

[22] Zhu H, Cao Z, Lian L, Ye G, Gao H, Wu J. CariesNet: a deep learning approach for segmentation of multi-stage caries lesion from oral panoramic X-ray image. Neural Comput Applic. 2022;35:16051–9. 10.1007/s00521-021-06684-2 PMC8736291 35017793

[23] Ying S, Wang B, Zhu H, Liu W, Huang F (2022). Caries segmentation on tooth X-ray images with a deep network. J Dent..

